# Patient characteristics and telehealth are associated with attendance rates in an outpatient rehabilitation infant bridge program

**DOI:** 10.1371/journal.pone.0301219

**Published:** 2024-03-27

**Authors:** Tiana T. Nguyen, Sang S. Pak, Matthew J. Miller

**Affiliations:** 1 Department of Occupational Therapy, University of California, San Francisco, San Francisco, California, United States of America; 2 Department of Occupational Therapy, Samuel Merritt University, Oakland, California, United States of America; 3 Department of Physical Therapy and Rehabilitation Science, University of California, San Francisco, San Francisco, California, United States of America; Istanbul University Istanbul Faculty of Medicine: Istanbul Universitesi Istanbul Tip Fakultesi, TURKEY

## Abstract

**Objective:**

To describe the characteristics of patients who received outpatient therapy services through an infant bridge program using telehealth mode of service delivery and to identify if attendance rates vary by mode of service delivery. We hypothesized that telehealth visits will increase attendance rates.

**Design:**

Retrospective, cross-sectional study.

**Setting:**

UCSF Benioff Children’s Hospital outpatient infant bridge program.

**Participants:**

Eighty infants with a history of NICU admission and scheduled for a therapy appointment between June 1, 2019 and December 31, 2020 were included in the study. Participants had an average(SD) gestational age of 34.63(4.41) weeks and length of stay was 43.55(56.03) weeks. The majority were English-speaking (96.3%), White (37.5%), and had commercial insurance (72.5%).

**Main outcome measure:**

Descriptive analyses were conducted across the entire group along with service delivery model subgroup analysis. Logistic regression was performed to assess patient characteristics associated with attendance and if service delivery model influences attendance.

**Results:**

In the analysis of 596 scheduled visits, there were more completed telehealth sessions than for in-person sessions (90.0% versus 84.1%, *p* = .011). For in-person sessions, infants (N = 40) with lower birth gestational ages (*p* = .009), longer length of stay (*p* = .041), and Medi-Cal insurance (*p* = .006) were more likely to have ≥2 missed appointments. For the telehealth sessions, infants (N = 40) who had longer length of stay (*p* = .040) were more likely to have ≥2 missed appointments. There is a higher likelihood of ≥2 missed appointments for patients with a longer length of stay (OR = 1.02, 95% CI [1.01, 1.03]) and for in-person service delivery when compared to telehealth (OR = 6.25, 95% CI [1.37, 28.57]).

**Conclusions:**

Telehealth was associated with higher likelihood of attendance, revealing that telehealth has the potential to increase access to early therapy services for certain populations. Future studies with larger sample sizes to determine which populations benefit from telehealth is recommended.

## Introduction

One in 10 infants are born prematurely in the United States and preterm infants have higher rates of developmental delays and disabilities [1]. Developmental disabilities may include breathing problems, feeding difficulties, cerebral palsy, developmental delay, vision problems, and hearing problems [1]. Early rehabilitative services (i.e., occupational and physical therapies) that start in the neonatal intensive care unit (NICU) are used to mitigate the potential risks of developmental disabilities among preterm infants.

Publicly funded early intervention programs that provide rehabilitative services for preterm infants who are discharged from the NICU effectively improve developmental and health outcomes for infants and families [2]. For example, early intervention rehabilitative services that include developmental interventions for NICU graduates were found to positively influence the development of motor and cognitive skills when compared to infants who did not receive early intervention rehabilitative services [3–5]. Parents who participated in early intervention rehabilitative services also felt more confident in caring for their infants, helping their infants progress with their developmental milestones, and developing coping strategies when compared to parents who did not receive rehabilitative services [6]. Higher volume of early intervention rehabilitative services also leads to greater functional gains, where an additional hour of rehabilitative services a month was associated with a 3-point increase in the social-emotional, cognitive, and adaptive domains on the Child Outcomes Summary [7].

Attending appointments is critical to receiving a higher volume of rehabilitative services. While a lower volume of rehabilitation services may be less effective in making functional gains, there are additional health service delivery consequences to missing rehabilitation appointments. The downstream effect of missed appointments includes wasted clinical time and resources resulting in an opportunity cost for other patients that could have used those services [8, 9]. Further, this places a burden on the administrative processes and resources, as the patient needs to be contacted and rescheduled, increasing the marginal cost for the clinic.[10] Despite these clinical challenges, difficulty with transportation, inconvenient locations, financial resources, and employer benefits (i.e., maternity leave, flexible time off, insurance coverage) all influence a families’ ability to attend NICU follow-up clinic and therapy appointments [11, 12].

Telehealth is a potential service delivery method that could be used to address barriers to receiving rehabilitation services during the transition from the NICU to home for preterm infants [13]. Telehealth eases the time and financial burdens of traveling to the clinic, potentially increasing access to therapy services for those living in underserved and rural area [14–17]. Telehealth has also improved attendance rates in NICU follow-up clinics and pediatric occupational therapy settings [13, 18]. However, the influence of telehealth for early rehabilitative services for NICU graduates has not been explored. Therefore, the purpose of this study was to determine if rehabilitation service attendance among NICU graduates differed among those with in-person or telehealth service delivery model. We also wanted to identify patient characteristics that were related to rehabilitation attendance. We hypothesize that telehealth visits were associated with higher attendance rates. This data is used to help guide which patient characteristics may benefit from in-person and/or telehealth services.

## Method

### Study design, site, participants, and data sources

We conducted a retrospective analysis of electronic health records data from infants who had a history of NICU admission and were scheduled for an appointment in our University of California, San Francisco Benioff’s Children Hospital (UCSF BCH) infant bridge program between June 1, 2019 and December 31, 2020. The data from the electronic health records was accessed between November 24, 2021 and December 1, 2021. The outpatient rehabilitation therapy team at UCSF BCH provides short-term outpatient physical and/or occupational therapy to bridge the gap in services between discharge home from the NICU to initiation of early intervention services offered through the state, which can take up to 6 months. In March 2020, UCSF BCH infant bridge program offered telehealth therapy services during the COVID-19 pandemic and has continued to offer these services to address ongoing challenges with the pandemic and patient and family barriers to accessing care.

We included all infants with a history of NICU admission and who received UCSF BCH bridge therapy services between June 1, 2019 and December 31, 2020. Infants with appointments between June 1, 2019 to March 30, 2020 were seen for in-person sessions, and infants with appointments between April 1, 2020 and December 31, 2020 were seen for telehealth sessions. This study, including a waiver of consent, was reviewed and approved by the University of California, San Francisco Institutional Review Board.

### Measures

*Rehabilitation attendance* was our primary measure of interest and was categorized as participants having <2 or ≥ 2 missed appointments. We selected two or more missed appointments as a threshold for our analysis because our clinic policy indicates this is the number of missed appointments (i.e., “no-show” or cancel <24 hours’ notice) when patients will need a new referral for continued services.

*Sociodemographic data* included preferred language (English/Spanish), patient-identified race/ethnicity (Asian; White; Latinx; Black, Multi-Race/Ethnicity, and Other), sex assigned at birth (male/female), and gestational age (weeks). The number of participants in race/ethnicity categories of Black, Multi-Race/Ethnicity, and Other were combined into one variable due to small sample size.

*Clinical data* included visit type (in-person/telehealth), hospitalization length of stay (days), insurance (commercial/Medi-Cal; where Medi-Cal is California’s Medicaid healthcare program), medical complexity, rehabilitation provider type per session (occupational and/or physical therapy), and date of services. We extracted the visit type to ensure that those who were seen after April 1, 2020, received services using telehealth. Medical complexity was categorized as complex chronic disease, non-complex chronic disease, or without chronic disease using the Pediatric Medical Complexity Algorithm (PMCA) [19]. The PCMA is a validated tool that uses ICD-10 codes, number of body systems involved, and whether the disease is progressive to categorize medical complexity.

### Data analysis

First, we tested for differences in the distribution of the number of completed and missed appointments by in-person and telehealth using independent samples *t*-tests. Next, we used Pearson’s chi-squared and independent samples *t*-tests to assess differences in service delivery models (in-person versus telehealth) by rehabilitation attendance, sociodemographic characteristics (preferred language, patient-identified race/ethnicity, sex assigned at birth, gestational age), and clinical characteristics (hospitalization length of stay, insurance, medical complexity, rehabilitation provider type). We then stratified the participants into service delivery model (in-person and telehealth) to assess if there were differences in the number of missed appointments groups (<2 or ≥ 2 missed appointments) by sociodemographic characteristics (preferred language, patient-identified race/ethnicity, sex assigned at birth, gestational age), and clinical characteristics (hospitalization length of stay, insurance, medical complexity, rehabilitation provider type) by service delivery model. Finally, we used backwards stepwise logistic regression to identify patient characteristics that were associated with ≥2 missed appointments (*p* < .050). In our first step, we generated a full model with all predictor variables that had bivariate associations with attendance at a statistical significance of *p* < .250. Multi-collinearity among the predictor variables in the full model was tested using Spearman’s Rho to minimize risk for bias. We proceeded with backward elimination because none of the variables were highly correlated (R>0.70). In each cycle of backward elimination, the variable in the model with the largest *p*-value was removed. This iterative cycle continued until the final model only had predictor variables with a significance value of *p* < .100. IBM Statistical Product and Service Solution (SPSS) 28 was used for data management and analysis.

## Results

### Participants and rehabilitation utilization

**Fig 1:**
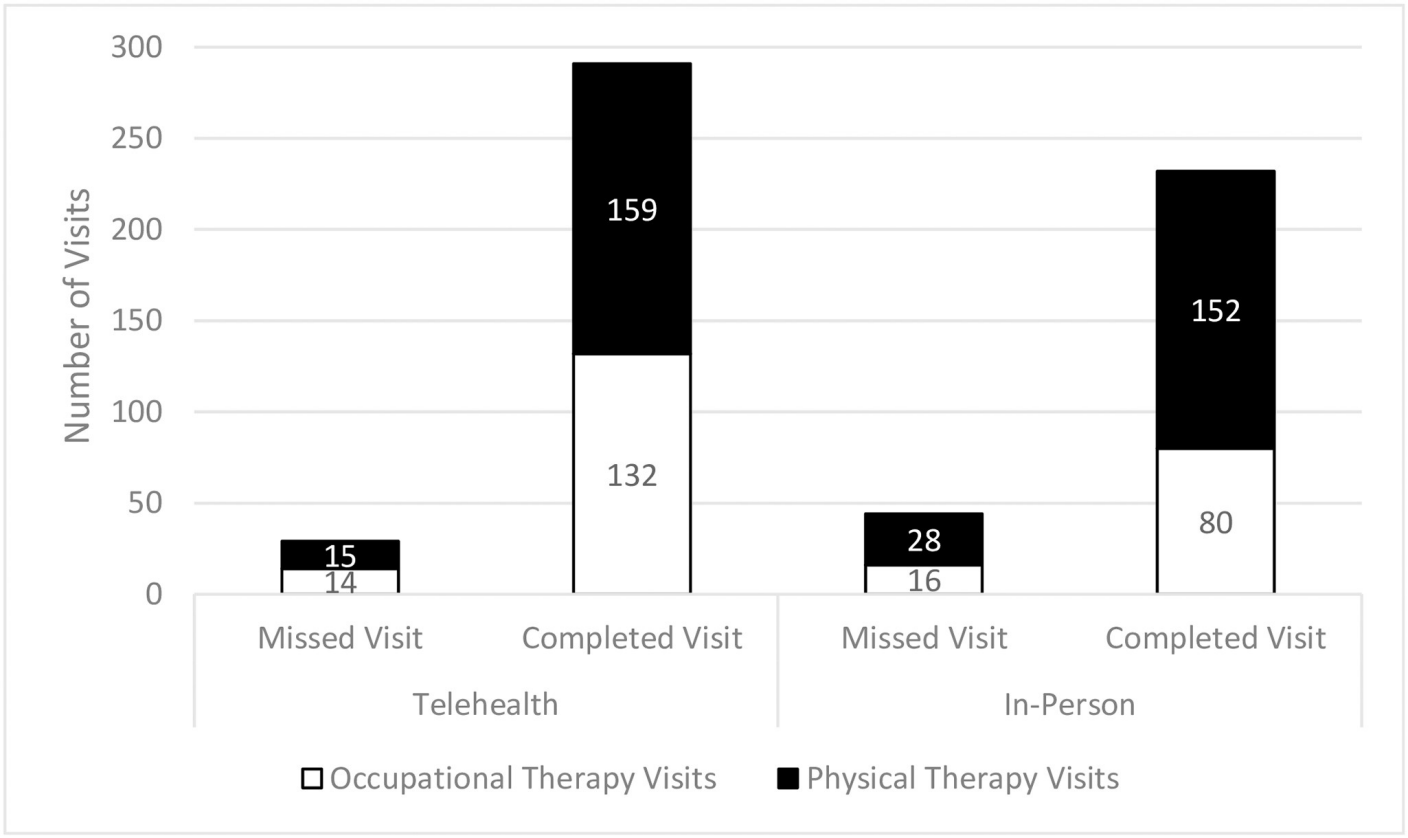
The number of completed and missed occupational and physical therapy visits at UCSF BCH infant bridge program by in-person and telehealth sessions.

Between June 1, 2019 and December 31, 2020, there were 80 infants who were provided rehabilitation services in the UCSF BCH infant bridge program (N = 40 in-person, N = 40 telehealth) and 596 scheduled visits. Infants seen in the program had an average (SD) gestational age of 34.63 (4.41) weeks and length of stay was 43.55 weeks (56.03), and the majority were English-speaking (96.3%), White (37.5%), and had commercial insurance (72.5%). The proportion of completed versus scheduled sessions was greater for telehealth compared to in-person (90.0% versus 84.0%, *p* = .011) (Fig 1). There were no differences in rehabilitation attendance, sociodemographic characteristics, or clinical characteristics between infants seen in-person or telehealth (Table 1).

**Table 1:** Participant characteristics for infants seen by the UCSF BCH infant bridge program and by in-person and telehealth sessions.

Participant Characteristics	Overall (n = 80)	In-Person (n = 40)	Telehealth (n = 40)	*p*-value
Preferred Language^a^				.077
English	77 (96.3%)	37 (92.5%)	40 (100.0%)	
Spanish	3 (3.8%)	3 (7.5%)	0 (0.0%)	
Patient-Identified Race/Ethnicity^a^				.726
White	30 (37.5%)	14 (35.0%)	16 (40.0%)	
Asian	22 (27.5%)	10 (25.0%)	12 (30.0%)	
Latinx	16 (20.0%)	10 (25.0%)	6 (15.0%)	
Black/African American, Multi-Race/Ethnicity, and Other	12 (15.0%)	6 (15.0%)	6 (15.0%)	
Sex Assigned at Birth^a^				.070
Male	46 (57.5%)	19 (47.5%)	27 (67.5%)	
Female	34 (42.5%)	21 (52.5%)	13 (32.5%)	
Gestational Age (Weeks) ^b^	34.63±4.41	35.33±4.21	33.92±4.55	.157
Length of Stay (Days) ^b^	43.55±56.03	36.48±52.67	50.63±59.02	.261
Insurance^a^				.133
Commercial	58 (72.5%)	26 (65.0%)	32 (80.0%)	
Medi-Cal	22 (27.5%)	14 (35.0%)	8 (20.0%)	
Medical Complexity^a^				.615
Complex Chronic Disease	23 (28.7%)	11 (27.5%)	12 (30.0%)	
Non-Complex Chronic Disease	17 (21.3%)	7 (17.5%)	10 (25.0%)	
Without Chronic Disease	40 (50.0%)	22 (55.0%)	18 (45.0%)	
Rehabilitation Provider Type^a^				.865
Occupational Therapy (OT)	25 (31.3%)	12 (48.0%)	13 (52.0%)	
Physical Therapy (PT)	37 (46.3%)	18 (48.6%)	19 (51.4%)	
Both OT & PT	18 (22.5%)	10 (55.6%)	8 (44.4%)	
Attendance^a^				.056
< 2 Missed Appointments	63 (78.8%)	28 (70.0%)	35 (87.5%)	
≥ 2 Missed Appointments	17 (21.3%)	12 (30.0%)	5 (12.5%)	

*Note*. Data presented as N(%) or mean±SD.

^a^Pearson’s chi-squared test.

^b^Independent samples t-test.

Overall, infants with ≥2 missed appointments, when compared to those with <2 missed appointments, had lower gestational ages (32.24 weeks versus 35.27 weeks, *p* = .011), longer lengths of stay (82.41 days versus 33.06 days, *p* = .021), and Medi-Cal insurance (52.9% versus 20.6%, *p* = .080). Similarly, infants with ≥2 in-person missed appointments had a lower gestational birth age (32.75 weeks versus 36.43 weeks, *p* = .009), longer length of stay (74.67 days versus 20.11 days, *p* = .041), and Medi-Cal insurance (66.7% versus 21.4%, *p* = .006). For the telehealth service delivery model, infants with ≥2 missed appointments only had a longer length of stay (101.00 days versus 43.43 days, *p* = .040), where no differences were noted for gestational age, length of stay, or insurance type (Table 2).

**Table 2:** Comparison of participant characteristics for infants who had <2 or ≥2 missed appointments at UCSF BCH infant bridge program between in-person and telehealth delivery.

Participant Characteristics	Overall			In-Person			Telehealth		
	< 2 Missed Appointments (n = 63)	≥ 2 Missed Appointments (n = 17)	*p*-value	< 2 Missed Appointments (n = 28)	≥ 2 Missed Appointments (n = 12)	*p*-value	< 2 Missed Appointments (n = 35)	≥ 2 Missed Appointments (n = 5)	*p*-value
Preferred Language^a^			.359			.238			.000*
English	60 (95.2%)	17 (100.0%)		25 (89.3%)	12 (100.0%)		35 (100.0%)	5 (100.0%)	
Spanish	3 (4.8%)	0 (0.0%)		3 (10.7%)	0 (0.0%)		0 (0.0%)	0 (0.0%)	
Patient-Identified Race/Ethnicity^a^			.099^+^			.460			.242
White	25 (39.7%)	5 (29.4%)		11 (39.3%)	3 (25.0%)		14 (40.0%)	2 (40.0%)	
Asian	20 (31.7%)	2 (11.8%)		8 (28.6%)	2 (16.7%)		12 (34.3%)	0 (0.0%)	
Latinx	11 (17.5%)	5 (29.4%)		6 (21.4%)	4 (33.3%)		5 (14.3%)	1 (20.0%)	
Black/African American, Multi-Race/Ethnicity, and Other	7 (11.1%)	5 (29.4%)		3 (10.7%)	3 (25.0%)		4 (11.4%)	2 (40.0%)	
Sex Assigned at Birth^a^			.326			.629			.702
Male	38 (60.3%)	8 (47.1%)		14 (50%)	5 (41.7%)		24 (68.6%)	3 (60%)	
Female	25 (39.7%)	9 (52.9%)		14 (50%)	7 (58.3%)		11 (31.4%)	2 (40%)	
Gestational Age (Weeks)^b^	35.27±4.24	32.24±4.32	.011*^+^	36.43±3.89	32.75±3.91	.009*	34.34±4.33	31.00±5.48	.126
Length of Stay (Days)^b^	33.06±43.75	82.41±77.78	.021*^+^	20.11±20.28	74.67±80.96	.041*	43.43±53.99	101.00±74.57	.040*
Insurance^a^			.008*^+^			.006*			1.000
Commercial	50 (79.4%)	8 (47.1%)		22 (78.6%)	4 (33.3%)		28 (80.0%)	4 (80.0%)	
Medi-Cal	13 (20.6%)	9 (52.9%)		6 (21.4%)	8 (66.7%)		7 (20.0%)	1 (20.0%)	
Medical Complexity^a^			.230^+^			.716			.062
Complex Chronic Disease	18 (28.6%)	5 (29.4%)		8 (28.6%)	3 (25.0%)		10 (28.6%)	2 (40.0%)	
Non-Complex Chronic Disease	11 (17.4%)	6 (35.3%)		4 (14.3%)	3 (25.0%)		7 (20.0%)	3 (60.0%)	
Without Chronic Disease	34 (54.0%)	6 (35.3%)		16 (57.1%)	6 (50.0%)		18 (51.4%)	0 (0.0%)	
Rehabilitation Provider Type^a^			.096^+^			.054			.326
Occupational Therapy (OT)	20 (31.7%)	5 (29.4%)		10 (35.7%)	2 (16.7%)		10 (28.6%)	3 (60.0%)	
Physical Therapy (PT)	32 (50.8%)	5 (29.4%)		14 (50.0%)	4 (33.3%)		18 (51.4%)	1 (20.0%)	
Both OT & PT	11 (17.5%)	7 (41.2%)		4 (14.3%)	6 (50.0%)		7 (20.0%)	1 (20.0%)	

*p < .050

^+^Variable included in full model for backward elimination.

*Note*. Data presented as N(%) or mean±SD.

^a^Pearson’s chi-squared test.

^b^Independent samples t-test.

Six variables had bivariate associations ≥2 missed appointments (*p* < .250) and were included in our full model for backward logistic regression: race/ethnicity, gestational age, length of stay, insurance, medical complexity, type of therapy, and service delivery model (Table 2). Upon completion of iterative backward elimination of variables, the odds (OR, 95% CI) of ≥2 missed appointments was greater for patients with a longer length of stay (OR = 1.02, 95% CI [1.01, 1.03]) and for in-person service delivery when compared to telehealth (OR = 6.25, 95% CI [1.37, 28.57]) (Table 3).

**Table 3:** Results of backward elimination in variables that predict ≥2 missed appointments.

	Model Statistics		Predictor Statistics	
Variable	Nagelkerke R Square	Chi-Square	Odds Ratio [95% CI]	*p*-value
Final Model	.29	16.21^+^		< .001*
Length of Stay (Days)			1.02 [1.01–1.03]	.002*
In-Person Service Delivery Model^a^			6.25 [1.37–28.57]	.018*

*p < .050

^+^The chi-square statistic for the final model was significant (*p* < .001)

^a^Reference group: Telehealth service delivery model

## Discussion

The purpose of this study was to determine if rehabilitation service attendance among NICU graduates differed among those with in-person or telehealth service delivery model. We identified that 91.0% of scheduled bridge appointments were completed, compared to 84.0% of in-person appointments. We also aimed to identify sociodemographic and clinical characteristics that were associated with rehabilitation session attendance. First, we identified that lower gestational age, longer length of stay and Medi-Cal insurance were associated with missing ≥2 in-person sessions, whereas only longer length of stay was associated with missing ≥2 telehealth sessions. Our final analysis indicated that longer length of stay and in-person sessions were associated with missing ≥2 sessions in our UCSF BCH infant bridge program. These findings can be used to inform future decisions in which service delivery models for outpatient therapy services best address our patients’ needs.

The first objective of this study was to describe the characteristics of patients who received outpatient therapy services through the UCSF BCH infant bridge program using telehealth mode of service delivery. There were no significant differences in the characteristics of participants who had in-person visits and those who had telehealth visits. On the contrary, a previous study in a pediatric neurology setting found telehealth use differed among patients living in rural areas by insurance types and other sociodemographic characteristics.(20) Dayal et al.(20) also reported that patients who use telehealth had different neurological diagnoses and fewer complex chronic conditions, whereas we found that our participants who had in-person visits when compared to telehealth visits who had complex chronic diseases were similar (27.5% versus 30.0%, *p* = .615). While our sample characteristics may differ from prior work because of the populations served, further research is needed to determine if study findings vary because of access patterns that emerged during the COVID-19 pandemic.

The second objective of this study was to identify which patient characteristics influence rehabilitation session attendance and if attendance differs by mode of service delivery. We confirmed our hypothesis and found that telehealth is associated with fewer missed rehabilitation session appointments. This is consistent with previous studies’ findings for other outpatient practice settings [9, 20, 21]. Childs et al. [21] found that appointment attendance was greater for both adolescents (75.6%) and adults (72.2%) who received telehealth versus in-person services in a psychiatric intensive outpatient program. Dayal et al. [20] found that patients who received telehealth versus in-person pediatric outpatient neurology care were more likely to attend their visits (OR = 1.46, 95% CI [1.27–1.68]). Finally, Snoswell & Comans [9] found a 68.0% reduction in missed appointments with an odds ratio of 0.32 (p<0.002) for patients who received telehealth versus in-person in an immunology outpatient clinic.

Participants who had longer length of stays have greater odds of ≥2 missed appointments (OR 1.02, 95% CI [1.01, 1.03]). Importantly, there were differences in the mean length of stays by missed appointment status for both in-person (20.11 versus 74.67, *p* = .041) and telehealth (43.43 versus 101.00, *p* = .040) groups. These findings are different than prior studies which identified that longer lengths of stay were associated with attendance in NICU follow-up appointments [22, 23]. Specifically, longer lengths of stay were associated with greater odds of attending their high-risk follow-up clinic appointments at the corrected age of 18–24 months [23] and neonatal neurodevelopmental follow-up clinic [22]. Our study findings may be different because of differences in practice settings and purpose of the visit. Swearingen et al. [22] and Kim et al. [23] looked specifically at high-risk infant follow up clinics, in which all infants who meet their criteria would get follow up with a multi-disciplinary developmental team at specific time points (usually every 6 months). This difference may also be due to a difference in patient population as Swearingen et al. [22] studied infants who were born at less than <33 weeks gestation and Kim et al. [23] looked at very low birth weight infants (<1500 grams). These studies attributed their findings to that infants with shorter length of stay are usually older in birth gestational age and less medically complex, therefore, parents may have less concerns about their infant’s development. In our present study of the UCSF BCH infant bridge program, it is possible that families of infants who have longer length of stays may be burned out from prolonged therapy services in the hospital or that they may have more appointments to manage that can either cause them to forget or need to re-prioritize their appointments, but further research is needed determine the cause of missed appointments.

We also found that there was a significant difference between the means of gestational ages and insurance types for attendance rates. Participants with a lower gestational age were associated with ≥2 missed appointments, specifically in the in-person group (36.43 days versus 32.75 days, *p* = .009). Similarly, Kim et al. [23] found that the follow up rates were inversely related to gestational age. On the contrary, Swearingen et al. [22] found that older gestational age was associated with not attending NICU follow-up appointments. This may be due to differences in patient population and practice setting as stated previously. We also found that participants who have public (Medi-Cal) insurance had significantly more ≥2 missed appointments than participants who have private insurance for the in-person group (57.1% versus 15.4%, *p* = .006) but not for the telehealth group (12.5% versus 12.5%, *p* = 1.000), therefore showing a potential disparity. These findings suggest that telehealth may be an option to address barriers to attendance for infants with lower gestational ages and for people who have Medi-Cal insurance.

We found that race/ethnicity was also not associated with attendance. However, small sample size with limited representation of Black and Multi-Race/Ethnicity was a limitation in this study and had to be combined into one group. Childs et al. [21] found that telehealth improved attendance across all racial and ethnic groups for psychiatric services. However, Walters et al. [24] found that there are racial disparities in telemedicine utilization, as Black patients used telemedicine less frequently than non-Black patients. Swearingen et al. [22] and Pai et al. [25] found that African American race was associated with not attending NICU follow-up appointments. Clinicians should carefully assess if telehealth may be beneficial for preterm infants who may need multisystem evaluation and rehabilitation. Future studies should include a larger sample size including a more racially and ethnically diverse sample and different preferred languages.

### Study limitations

We included data from a relatively small sample of patients at one site, potentially limiting our analytic power and generalizability of our findings to other contexts and populations. We also used data from the initial stages of the COVID-19 pandemic when public policies and telehealth service delivery were changing rapidly. Future studies with larger sample sizes and across multiple sites should be considered to determine if there is an impact on attendance when patients and clinicians collaborate to choose which service delivery model best meets their needs.

## Conclusion

Telehealth was associated with higher odds of rehabilitation session attendance to an infant bridge program. Thus, telehealth could be a feasible method to target attendance barriers, especially for those with longer hospital length of stay. These findings should be used to guide future research, policy, and clinical efforts to improve the quality of rehabilitative care for NICU graduates.

## Supporting information

S1 Checklist STROBE statement—Checklist of items that should be included in reports of observational studies. (DOCX)

## Abbreviations

UCSF BCH: UCSF Benioff’s Children Hospital
NICU: neonatal intensive care unit
